# Proceedings of the 5^th^ Ibero-American Symposium on Sport and Physical Activity: Nutrition & Training (SIDANE) and 1^st^ International Conference on Nutrition Applied to Physical Activity and Sport

**DOI:** 10.1186/s12919-024-00288-z

**Published:** 2024-02-06

## Edited by Diego A. Bonilla^1^, Alejandra Agudelo-Martínez^2^

### ^1^Research Division, DBSS International SAS, Bogotá, Colombia; ^2^CESNUTRAL, Universidad CES, Medellín, Colombia

#### **Correspondence:** Diego A. Bonilla (dabonilla@dbss.pro); María A. Agudelo-Martínez (magudelo@ces.edu.co)

*BMC Proceedings 2024*, **18(Suppl 4)**

Exercise science and sports nutrition, as applied sciences, facilitate the generation of new knowledge and its application to contribute benefits to society, such as improving aspects of health and well-being in different populations. Despite the publication and dissemination of these advancements through various means, the rapid progress of science brings with it a questionable quality of information that is circulated. Therefore, a higher degree of rigor and contextualization is necessary. The 5^th^ Ibero-American Symposium on Sport and Physical Activity: Nutrition & Training (SIDANE) and the I° International Conference on Nutrition Applied to Physical Activity and Sport (CINAAD) serve as an academic platform for disseminating and presenting scientific advancements in exercise science and nutrition applied to physical performance and health.

The 5^th^ SIDANE and I° CINAAD was organized and hosted by the Faculty of Nutrition and Food Sciences at Universidad CES in partnership with the Research Division at Dynamical Business & Science Society - DBSS International SAS in Medellín, Colombia on 21 – 23 September 2023. Post-conference workshops occurred from September 24^th^ to October 1^st^ at Universidad CES. The event involved 20 researchers from Colombia, Mexico, the USA, Italy, the Czech Republic, Costa Rica, Spain, and Argentina, creating a genuinely interdisciplinary and translational scientific gathering with approximately 300 registered participants.

For three days, the event featured keynote presentations by invited speakers, oral presentations by young researchers, and highly anticipated poster sessions by students (all abstracts underwent peer review). Adding to the excitement of the event, the Scientific Committee recognized the contributions to the exercise and sports nutrition field made by Ruth Gallo, Rocío Gamez and Olga Quiroz (Pioneers in sports nutrition in Colombia by Universidad CES), Prof. Roberto Cannataro (DBSS Senior Researcher Award), MSc. Leidy T. Duque (DBSS Young Researcher Award), and presented certificates to PT. Elkin Lopera (NSCA-CPT Certification) and students from the International Master on Personal Training (DBSS Certification).

We would like to express our gratitude to all DBSS members and the staff and students from Universidad CES. Additionally, we extend our thanks to all researchers and students who embraced the opportunity to take part in this event, presenting their latest data, sharing their expertise, and actively contributing to transforming it into a genuinely interdisciplinary conference.

Sponsorship: Publication charges for this supplement were funded by the Research and Innovation Coordination at Universidad CES.

Organizers: Dynamical Business & Science Society—DBSS International SAS and the Faculty of Nutrition and Food Sciences at Universidad CES

Acknowledgements: The conference received funding from ‘Colombian Institute of Educational Credit and Technical Studies Abroad’ (ICETEX) and private sponsors/collaborators.

**Table 1:** Research Topics and Scientific Team at 5^th^ SIDANE and I° CINAAD

Research Topics covered at 5th SIDANE & I° CINAAD	Scientific Committee & Speakers	Evaluation Committee
Body composition	Diego A. Bonilla (Colombia)	Juan F. Córdoba (Colombia)
Physical performance	Piedad Roldan (Colombia)	Angélica Muñoz (Colombia)
Physical Rehabilitation	María A. Agudelo (Colombia)	Diego Morales (Argentina)
Nutrition and supplementation	Richard B. Kreider (USA)	María A. Agudelo (Colombia)
	Francisco Esparza (España)	Juan Carlos Granados (Colombia)
	Jana Kočí (República Checa)	Fernando Alacid (España)
	Erika Cione (Italia)	Christian Pureco (México)
	Katherine Franco (Colombia)	José Moncada (Costa Rica)
	José Moncada (Costa Rica)	Luis Felipe Bedoya (Colombia)
	Roberto Cannataro (Italia)	Mercedes Mora (Colombia)
	Faiber Jaramillo (Colombia)	Santiago Gómez (Colombia)
	Fernando Pérez Mesa (México)	Roberto Cannataro (Italia)
	David Cardona (Colombia)	Diego A. Bonilla (Colombia)
	Jeffrey R. Stout (USA)	Salvador Vargas (España)
	Christian Pureco (México)	Andrés Rojas (Colombia)
	Jorge Luis Petro (Colombia)	Jorge Luis Petro (Colombia)
	Maximiliano Kammerer (Colombia)	Gustavo Humeres (Argentina)
	Salvador Vargas (España)	Daniel Rojas (Costa Rica)
	Luis Felipe Bedoya (Colombia)	
	Ángel Rodríguez (México)	
	Juan Carlos Granados (Colombia)	
	Jorge Mario Vélez (Colombia)	

## Oral presentations

### O1 - Potential microRNA network modulating training adaptations following nutritional supplementation: a bioinformatics-assisted review

#### Alejandro Gómez-Pedraza^1,2^, Carlos A. Orozco^3^, Yurany Moreno^1^, Roberto Cannataro^4^, Richard B. Kreider^5^, Jorge L. Petro^1,6^, Diego A. Bonilla^1,6^

##### ^1^Research Division, Dynamical Business & Science Society – DBSS International SAS, Bogotá, Colombia; ^2^Bioquímica, Universidad Antonio Nariño, Bogotá, Colombia; ^3^Translational Research Group in Oncology and Cancer Biology, Instituto Nacional de Cancerología, Bogotá, Colombia; ^4^Galascreen Laboratories, Department of Pharmacy Health and Nutritional Sciences, University of Calabria, Rende, Italy; ^5^Exercise & Sport Nutrition Laboratory, Human Clinical Research Facility, Texas A&M University, College Station, USA; ^6^Research Group in Physical Activity, Sports and Health Sciences (GICAFS), Universidad de Córdoba, Montería, Colombia

###### **Correspondence:** Diego A. Bonilla (dabonilla@dbss.pro)

*BMC Proceedings 2024*, **18(Suppl 4)**O1

**Introduction:** MicroRNAs (miRNAs) are important biological elements that regulate and participate in the complexity of gene regulatory networks and their impact on various physiological adaptation processes [1]. Considering that a change in the expression of different miRNAs has been observed after nutritional supplementation [2], the objective of this bioinformatics-assisted review (BaR) was to explore a possible network of microRNAs responsible for optimizing adaptations to physical exercise. **Methods:** The BaR approach was followed according to previous research [3]. The search in the mirPub, PubMed/MEDLINE, Science Direct, and Google Scholar databases was conducted using the Boolean algorithm (miRNA or myoRNA or microRNA or miR) AND supplementation AND nutrition* AND “skeletal muscle” NOT diseases (miRNA or myoRNA or microRNA or miR*) AND (Supplement* OR nutri*) AND exercise NOT diseases. Free terms “miRNA,” “supplementation,” and “exercise” were also used. Scientific articles reporting results in healthy humans and including safe and effective nutrients such as creatine monohydrate, caffeine, beta-alanine, nitrate, and whey protein were selected. **Results:** The search resulted in a total of 141 references; however, after removing duplicates (*n* = 8), excluding by title and abstract (*n* = 110), and evaluating the full text (*n* = 20), only 11 references that met the inclusion criteria were selected. A set of 27 miRNAs was identified, and our preliminary results indicate that miR-1, miR-23a, miR-499, and miR-23b converge as potential physiological regulators of adaptation processes following the administration of nutritional supplements during exercise programs. **Conclusions:** This study delves for the first time into identifying a potential network of miRNAs induced by nutritional supplementation that converge as key molecules in adaptations to physical exercise. However, further research is needed, considering that experimental designs and involved populations are heterogeneous concerning diet, sex, age, nutritional supplementation, and exercise prescription.

**References**

1. Winkle M, El-Daly SM, Fabbri M, Calin GA. Noncoding RNA therapeutics - challenges and potential solutions. *Nat Rev Drug Discov*. 2021; 20(8): 629-651.
2. D'Souza RF, Zeng N, Markworth JF, Figueiredo VC, Hedges CP, Petersen AC, et al. Whey Protein Supplementation Post Resistance Exercise in Elderly Men Induces Changes in Muscle miRNA’s Compared to Resistance Exercise Alone. *Front Nutr*. 2019; 6:91.
3. Bonilla DA, Kreider RB, Stout JR, Forero DA, Kerksick CM, Roberts MD, et al. Metabolic Basis of Creatine in Health and Disease: A Bioinformatics-Assisted Review. *Nutrients*. 2021; 13(4).

### O2 - Food guide for pre-training, pre-match, and post-match meals in the Professional Soccer Team Deportivo Cali

#### Miguel Alzate-Soto^1^, Sharik C. Enríquez-Muñoz^1^, Michelle R. Marín-Ríos^1^, Juan S. Muñoz^1^, Angélica Astudillo^2^, Gustavo Portela^2^

##### ^1^Institución Universitaria Escuela Nacional del Deporte, Santiago de Cali, Colombia; ^2^Asociación Deportivo Cali - Sede Pance, Andalucía, Cali, Valle del Cauca

###### **Correspondence:** Michelle R. Marín-Ríos (mrmarin@endeporte.edu.co)

*BMC Proceedings 2024*, **18(Suppl 4)**O2

**Introduction:** Effective food portion management is essential for athletes to achieve desired body changes and enhance performance [1]. A food guide delivers nutritional education to soccer players, advising them on optimal consumption timing and empowering informed dietary decisions. This study aimed to create an illustrated food portions guide tailored for professional soccer players at Colombian Category 1A. **Methods:** This cross-sectional study involved 36 male elite soccer players from Deportivo Cali, averaging 23.9 years. GPS Catapult devices were used to estimate energy expenditure over a month of training and official matches in the competitive season. Evidence-based nutritional recommendations were considered to customize different mealtimes based on energy expenditure and playing positions [2,3]. Illustrated menus were developed for each feeding time based on the nutritional information from the Colombian Food Composition Table. **Results:** The guide prioritizes supplying the necessary energy for optimal performance during each sports moment to avoid relative energy deficiency syndrome. Carbohydrates play a pivotal role as the primary energy substrate, resulting in significantly larger portions compared to other macronutrients. Portion modulation was influenced by mealtime and playing position considering changes in energy expenditure. Proteins are increased during recovery periods and when players experience lower energy expenditure, leading to variable and substantial food portions for each playing position. Conversely, lipids represent the smallest portions, emphasizing low-fat intake to preserve caloric load and nutrient absorption. **Conclusions:** Even though soccer is a team sport, personalized nutritional management proved crucial, considering diverse positions entailed varying effort levels and physical capacities. This new food guide is a valuable tool for implementing portion control in macronutrient distribution, aligning with sports goals and energy expenditure, and ensuring athlete adherence to nutritional plans.

**References**

1. Collins J, Maughan RJ, Gleeson M, Bilsborough J, Jeukendrup A, Morton JP, Phillips SM, Armstrong L, Burke LM, Close GL, Duffield R, Larson-Meyer E, Louis J, Medina D, Meyer F, Rollo I, Sundgot-Borgen J, Wall BT, Boullosa B, Dupont G, Lizarraga A, Res P, Bizzini M, Castagna C, Cowie CM, D’Hooghe M, Geyer H, Meyer T, Papadimitriou N, Vouillamoz M, McCall A. UEFA expert group statement on nutrition in elite football. Current evidence to inform practical recommendations and guide future research. *Br J Sports Med*. 2021; 55(8):416.
2. Mountjoy M, Ackerman KE, Bailey DM, Burke LM, Constantini N, Hackney AC, Heikura IA, Melin A, Pensgaard AM, Stellingwerff T, Sundgot-Borgen JK, Torstveit MK, Jacobsen AU, Verhagen E, Budgett R, Engebretsen L, Erdener U. 2023 International Olympic Committee’s (IOC) consensus statement on Relative Energy Deficiency in Sport (REDs). *Br J Sports Med*. 2023; 57(17): 1073-1097.
3. Hulton AT, Malone JJ, Clarke ND, MacLaren DPM. Energy Requirements and Nutritional Strategies for Male Soccer Players: A Review and Suggestions for Practice. *Nutrients*. 2022; 14(3): 657.

### O3 - Effects of glycerol plus sodium on the hydration status in Colombian elite male soccer players

#### Jhoann S. Pérez-Arévalo^1^, Ingrid L. Herrera-Pinzón^2^, Catalina Chica-García^2^, Ligia S. Guerrero-Orjuela^1^

##### ^1^Departamento de Nutrición Humana, Universidad Nacional de Colombia, Bogotá, Colombia; ^2^Departamento de Ciencias Aplicadas al Deporte (DCARF), Azul & Blanco Millonarios Fútbol Club, Bogotá, Colombia

###### **Correspondence:** Jhoann S. Pérez-Arévalo (jhsperezar@unal.edu.co)

*BMC Proceedings 2024*, **18(Suppl 4)**O3

**Introduction:** The current evidence in elite athletes suggests that glycerol (GLY) may reduce the occurrence of adverse effects due to fluid loss [1,2]. It is worth noting that GLY is classified in category A according to the Australian Institute of Sport (AIS) [3]. Although effects are promising, less information is available regarding the addition of sodium (Na^+^) to GLY [4]; thus, this study evaluated this combination on the hydration status of elite athletes trained at high altitude (2600 m.a.s.l.). **Methods:** A double-blinded randomized controlled clinical trial was performed in 21 Colombian elite male soccer players (24.4 [3.9] y), which were randomly allocated into Group A (control, *n* = 11) and Grupo B (intervention, *n* = 10). The glycerol solution was prepared according to AIS guidelines as 1.2 g GLY per kilogram of body mass (BM) but we added 25 mL per BM kilogram of a Na^+^ solution (600 mg/L). After breakfast, Group B received four 600-milliliter doses, spaced 15min apart, and 60min before a soccer training match. Primary outcomes included urine specific gravity (USG), percentage of BM loss, liquid volume ingested, excreted urine volume, [Na^+^] in sweat, and sweat rate at pre- and post-training. The [Na^+^] in sweat was estimated using ion-selective electrode technique with a patch on the dorsal mid-forearm and the expression: whole-body sweat [Na^+^] (mmol/L) = 0.57 (forearm sweat [Na^+^]) + 11.05. **Results**: Group A showed a significant reduction on USG (*P*=0.004) while Group B did not (*P*=0.677). Group B had a lower percentage of BM loss compared to group A (1.3% versus 1.7%, *P*=0.043). Sweat rate, excreted urine volume, and whole-body sweat [Na^+^] showed no significant changes in either group (*P*>0.05). The ingested liquid volume was higher in Group B players (*P*=0.017). **Conclusions**: Using GLY with added Na^+^ maintains hydration status in soccer players without changes on USG or whole-body sweat [Na^+^]. It also promoted a lower percentage of BM loss, potentially reducing loss of fluids.

**References**

1. Koehler K, Thevis M, Schaenzer W. Meta-analysis: Effects of glycerol administration on plasma volume, haemoglobin, and haematocrit. *Drug Test Anal*. 2013; 5(11-12): 896-9.
2. van Rosendal SP, Osborne MA, Fassett RG, Coombes JS. Guidelines for glycerol use in hyperhydration and rehydration associated with exercise. *Sports Med*. 2010; 40(2): 113-29.
3. McCubbin AJ, Allanson BA, Caldwell Odgers JN, Cort MM, Costa RJS, Cox GR, et al. Sports Dietitians Australia Position Statement: Nutrition for Exercise in Hot Environments. *Int J Sport Nutr Exerc Metab*. 2020; 30(1): 83-98.
4. Goulet EDB, De La Flore A, Savoie FA, Gosselin J. Salt + Glycerol-Induced Hyperhydration Enhances Fluid Retention More Than Salt- or Glycerol-Induced Hyperhydration. *Int J Sport Nutr Exerc Metab*. 2018; 28(3): 246-52.

### O4 - Plant-based diets in endurance athletes: a scoping review with dietary and nutritional recommendations

#### Juan D. Quintero^1^, Jovanny Fausto-González^1^, Iran A. Lomeli-Ulloa^2^, Mónica G. Reyes-Monroy^2^, Daniel Rojas-Valverde^3,4^, Jose M. Martínez-Sanz^5,6^, Jorge L. Petro^1,7^, Diego A. Bonilla^1,7^

##### ^1^Research Division, Dynamical Business & Science Society – DBSS International SAS, Bogotá, Colombia; ^2^Licenciatura en Nutrición, Unidad Académica de Salud Integral, Universidad Autónoma de Nayarit, Tepic, México; ^3^Núcleo de Estudios para el Alto Rendimiento y la Salud (CIDISAD-NARS), Escuela Ciencias del Movimiento Humano y Calidad de Vida (CIEMHCAVI), Universidad Nacional, Heredia, Costa Rica; ^4^Clínica de Lesiones Deportivas (Rehab&Readapt), Escuela Ciencias del Movimiento Humano y Calidad de Vida (CIEMHCAVI), Universidad Nacional, Heredia, Costa Rica; ^5^Food and Nutrition Research Group (ALINUT), University of Alicante, Alicante, Spain; ^6^Nursing Department, Faculty of Health Sciences, University of Alicante, Alicante, Spain; ^7^Research Group in Physical Activity, Sports and Health Sciences (GICAFS), Universidad de Córdoba, Montería, Colombia

###### **Correspondence:** Diego A. Bonilla (dabonilla@dbss.pro)

*BMC Proceedings 2024*, **18(Suppl 4)**O4

**Introduction:** Plant-Based Diets (PBD) are dietary patterns that exclude some or all animal-origin foods [1], with vegetarian and vegan diets being prevalent among endurance athletes [2-3]. This scoping review aims to explore emerging scientific evidence on PBD in endurance athletes and formulate practical dietary and nutritional recommendations. **Methods:** We adhered to the Preferred Reporting Items for Systematic Reviews and Meta-Analyses for Scoping Reviews (PRISMA-ScR) guidelines [4] and employed Boolean algorithms on PubMed/MEDLINE, Science Direct, and Google Scholar. Scoping reviews consider various types of evidence (quantitative and qualitative) and are particularly useful in emerging or rapidly evolving fields, such as PBD. Finally, a bibliometric analysis was performed using VOSviewer v1.6.19 software. **Results:** Initially, 1075 references were retrieved, reducing to 12 meeting inclusion criteria after eliminating duplicates (*n*=42), excluding by title and abstract (*n*=1009), and full-text assessment (*n*=13). Higher academic production and impact (citations) were noted in Germany, the United States, Austria, Switzerland, and Italy. PBDs showed higher total carbohydrate intake, attributed to elevated fiber consumption, and lower intake of proteins, saturated fatty acids, and cholesterol. Special attention is needed for potential deficiencies in vitamin B12, omega-3, iron, calcium, creatine, vitamin D, and zinc. Acknowledging study heterogeneity and limited research, we offer practical dietary-nutritional recommendations for endurance athletes and encourage sports dietitians and nutritionists to consider individual characteristics and perform regular nutritional assessments. **Conclusions:** Collective findings suggest PBDs may provide performance comparable to omnivorous diets in endurance athletes, emphasizing the need to address specific nutrient requirements. Further research is necessary to comprehend fully the long-term effects of PBDs on athletic performance associated with nutrient intake differences.

**Protocol registration**

PRISMA-ScR protocol: doi:10.6084/m9.figshare.24115992.v3

**References**

1. Hargreaves SM, Rosenfeld DL, Moreira AVB, Zandonadi RP. Plant-based and vegetarian diets: an overview and definition of these dietary patterns. *Eur J Nutr*. 2023; 62(3): 1109-1121.
2. Pelly FE, Burkhart SJ. Dietary regimens of athletes competing at the Delhi 2010 Commonwealth Games. *Int J Sport Nutr Exerc Metab*. 2014; 24(1): 28-36.
3. Wirnitzer K, Tanous D, Motevalli M, Wirnitzer G, Leitzmann C, Pichler R, Rosemann T, Knechtle B. Prevalence of Female and Male Vegan and Non-Vegan Endurance Runners and the Potential Associations of Diet Type and BMI with Performance-Results from the NURMI Study (Step 1). *Nutrients*. 2022; 14(18): 3803.
4. Tricco AC, Lillie E, Zarin W, O’Brien KK, Colquhoun H, Levac D, Moher D, Peters MDJ, Horsley T, Weeks L, Hempel S, Akl EA, Chang C, McGowan J, Stewart L, Hartling L, Aldcroft A, Wilson MG, Garritty C, Lewin S, Godfrey CM, Macdonald MT, Langlois EV, Soares-Weiser K, Moriarty J, Clifford T, Tunçalp Ö, Straus SE. PRISMA Extension for Scoping Reviews (PRISMA-ScR): Checklist and Explanation. *Ann Intern Med*. 2018; 169(7): 467-473.

### O5 - Relationship between phase angle and body composition in young Mexican university adults

#### Jorge A. Aburto-Corona^1^, Mateo Yepes-Castillo^1,2^, Juan J. Calleja-Núñez^1^, Luis M. Gómez-Miranda^1^, Roberto Espinoza-Gutiérrez^1^, Cindy J. Castro-Ramírez^2^

##### ^1^Grupo de Investigación UABC-CA-341 Rendimiento Físico y Salud, Facultad de Deportes, Universidad Autónoma de Baja California, Tijuana, México; ^2^Grupo de Investigación Cuerpo, Sujeto y Educación, Facultad de Cultura Física, Deporte y Recreación, Universidad de Santo Tomás, Bogotá, Colombia

###### **Correspondence:** Jorge A. Aburto-Corona (jorge.aburto@uabc.edu.mx)

*BMC Proceedings 2024*, **18(Suppl 4)**O5

**Introduction:** Body composition (BC) assessment allows monitoring the nutritional status and physical-academic performance of university students. High percentages of body fat (%BF) and low amounts of skeletal muscle mass (MM) are associated with several health problems. Complementary, phase angle (PhA) represents a variable associated with overall health (1,2), as it is a parameter of Bioelectrical Impedance Analysis (BIA) that describes cellular integrity and function. This study aimed to determine the relationship between PhA and BC-related variables estimated by BIA (total body water [TBW], intracellular water [ICW], extracellular water [ECW], %BF, and MM) in undergraduate students. **Methods:** A correlational cross-sectional study was conducted with a sample size of 69 subjects calculated a priori (alpha error of 0.05 and statistical power of 0.90) at the “Laboratorio de Biociencias de la Motricidad Humana” at Autonomous University of Baja California (Tijuana, Mexico). Stature (InBody BSM170) and the BIA (InBody 770) were measured according to ACSM guidelines. **Results**: A total of 77 apparently healthy Physical Education majors (46M; 31F; 21.7 [2.1] years; 166.4 [7.7] cm; 71.0 [18.5] kg) participated in this study. Data for TBW (37.7 [8.3] L), ICW (23.7 [5.3] L), ECW (14.0 [3.0] L), %BF (26.2 [9.1] %), MM (29.0 [7.0] kg), and PhA (6.3 [0.8] °) were analyzed. PhA showed a significant and moderate-to-high correlation with MM (*r*=0.713; *p*=0.001), TBW (*r*=0.689; *p*=0.001), ICW (*r*=0.714; *p*=0.001), and ECW (*r*=0.642; *p*=0.001). No significant relationship was found between PhA and %BF (*r*=-0.217; *p*=0.058). Conclusions: PhA correlates with MM as well as various components of body water. However, PhA is not associated with %BF in the studied population. Future research might study the changes on PhA under different hydration levels.

**References**

1. Streb AR, Hansen F, Gabiatti MP, Tozetto WR, Del Duca GF. Phase angle associated with different indicators of health-related physical fitness in adults with obesity. *Physiol Behav*. 2020; 225.
2. Lukaski HC, Talluri A. Phase angle as an index of physiological status: Validating bioelectrical assessments of hydration and cell mass in health and disease. *Rev Endocr Metab Disord*. 2022; 24(3): 371–379.

### O6 - Effects of carbohydrate mouth rinse on athletic performance: a systematic review

#### Mayra A. Márquez-Rodríguez^1^, Andrés F. Paipilla^2^, Johana Montoya^1^, John E. Giraldo-Vallejo^1^, Jorge L. Petro^1,3^, Alexandra Pérez-Idárraga^4^, Katherine Franco^5^, Richard B. Kreider^6^, Diego A. Bonilla^1,3,5^

##### ^1^Research Division, Dynamical Business & Science Society – DBSS International SAS, Bogotá, Colombia; ^2^Nutrición y Dietética, Facultad de Medicina, Universidad Nacional de Colombia, Bogotá, Colombia; ^3^Research Group in Physical Activity, Sports and Health Sciences (GICAFS), Universidad de Córdoba, Montería, Colombia; ^4^Move Nutrition, 050021 Medellin, Colombia; ^5^Grupo de Investigación NUTRAL, Facultad de Ciencias de Nutrición y Alimentos, Universidad CES, Medellín, Colombia; ^6^Exercise & Sport Nutrition Laboratory, Human Clinical Research Facility, Texas A&M University, College Station, USA

###### **Correspondence:** Diego A. Bonilla (dabonilla@dbss.pro)

*BMC Proceedings 2024*, **18(Suppl 4)**O6

**Introduction:** The ergogenic potential of mouth rinses with different types of carbohydrates ingested before or during physical exertion has been reported in both professional and recreational athletes [1-4]. However, context-dependent practical conclusions (sex, type of exercise, timing, fasting) are required. This review analyzes randomized clinical trials (RCTs) conducted to date to provide evidence-based recommendations for sports nutritionists and practitioners looking to incorporate this nutritional strategy to enhance athletes’ training adaptations. **Methods:** We followed the PRISMA guidelines and searched for RCTs published between 2008-2023 and available at Pubmed/MEDLINE, Science Direct, Ovid, Google Scholar, and PEDro databases using the Boolean algorithm *(Carbohydrates mouth rinsing OR carbohydrate mouth rinse) AND performance*. Risk of bias assessment for the RCTs was conducted using the RoB2.0 tool. **Results:** The results on 418 participants (77.5% men, 22.5% women) in 28 clinical trials that met the inclusion criteria were analyzed. The use of a 25 ml solution of mouth rinse with simple carbohydrates and a duration in the oral cavity between 5-10 seconds, consumed before and during a moderate-intensity repeated test lasting 25-60 minutes, appears to improve athletic performance. However, these results may vary due to fasting time, the type and intensity of exercise in endurance and strength/power events, and the physical condition of the subjects. **Conclusions:** The concentration of mouth rinse may not be a determining factor; however, the duration in the oral cavity could increase receptor stimulation. Furthermore, the effect is even greater when subjects are in a fasting state. Thus, including this strategy in low glycogen availability training could be suggested. Its association with high-intensity, short-duration modalities, such as improving maximum strength and decreasing declining motor activity, is still limited.

**Protocol registration**

PRISMA protocol: doi:10.6084/m9.figshare.24124638

**References**

1. Carter JM, Jeukendrup AE, Jones DA. The effect of carbohydrate mouth rinse on 1-h cycle time trial performance. Med Sci Sports Exerc. 2004;36(12):2107-11.
2. Kamaruddin HK, Ooi CH, Mundel T, Aziz AR, Che Muhamed AM. The ergogenic potency of carbohydrate mouth rinse on endurance running performance of dehydrated athletes. Eur J Appl Physiol. 2019;119(8):1711-23.
3. Maleklou Faezeh AZ. The effect of carbohydrate mouth rinsing on functional capacity of overweight women. Obesity Medicine. 2020;18.
4. Khong TK, Selvanayagam V, Yusof A. Effect of glucose and sodium chloride mouth rinses on neuromuscular fatigue: a preliminary study. Eur J Sport Sci. 2021;21(2):224-30.

### O7 - Effects of lactose before training on sports performance of professional Colombian young cyclists

#### Andrés C. Gómez-Álvarez^1^, Lady C. Portillo-Lozano^1^, Astrid E. Quinchía^2^, Erik Bejarano^2^, Camilo E. Povea-Combariza^2^, Ligia S. Guerrero-Orjuela^3^

##### ^1^Maestría en Nutrición Deportiva, Facultad de Ciencias de la Nutrición y los Alimentos, Universidad CES, Medellín, Colombia; ^2^Centro de Ciencias del Deporte, Ministerio del Deporte, Bogotá, Colombia; ^3^Departamento de Nutrición Humana, Universidad Nacional de Colombia, Bogotá, Colombia

###### **Correspondence:** Ligia S. Guerrero-Orjuela (leguerreroo@unal.edu.co)

*BMC Proceedings 2024*, **18(Suppl 4)**O7

**Introduction:** Milk is a nutrient-rich food [1] with the potential to enhance athletic performance due to its carbohydrate content (lactose), isotonic-like osmolarity, and relatively low cost [2–5]. This study assessed the impact of lactose-containing milk intake on the performance of highly trained cyclists at moderate altitude. **Methods:** A double-blind, crossover, randomized trial was conducted in eight young professional male cyclists. The initial test was done without any liquid intake (NB). Subsequent tests involved whole milk (WM), lactose-free milk (LFM), or plant-based beverage (PB). Participants received 600ml one hour pre-exercise, supplying 30g/h of carbohydrates (0.5g/min oxidation rate). Following two activations, the Functional Threshold Power protocol in a 20-min time trial (FTP_20_) was performed, measuring gas exchange and ventilatory variables. Blood glucose and lactate were assessed, along with perceived gastrointestinal effects. **Results:** First activation showed a statistically significantly higher work rate with LFM compared to PB (*P*=0.0024) and WM (*P*<0.001). Relative power was higher in PB and WM compared to LFM and NB. For the second activation, all beverages resulted in lower heart rate compared to NB. In the FTP_20_, WM and PB showed higher relative power (4.36 [0.25] W and 4.30 [0.22] W, respectively), and work rate was higher after all beverages than NB. The Training Stress Score (based on normalized power, intensity factor, and cycling time) was higher with all three beverages compared to NB. WM exhibited higher exercise ventilation (156.7 [14.4] L/min) and greater relative VO_2max_ (60.14 [4.92] ml/kg/min) than other beverages. No significant changes were observed in blood glucose and lactate. LFM induced more gastrointestinal symptoms (38.6%) than WM (22.7%) and PB (14.8%) (with abdominal distension [58.3%] being predominant). **Conclusions:** Lactose-containing milk improves training variables by ~0.6-8.3% during high-intensity efforts in a graded exercise test. Notably, any beverage intake led to gastrointestinal symptoms. Further research in elite athletes is necessary, allowing more time between tests and prior gastrointestinal training.

**References**

1. Lee JKW, Maughan RJ, Shirreffs SM, Watson P. Effects of milk ingestion on prolonged exercise capacity in young, healthy men. Nutrition. 2008 Apr;24(4):340–7.
2. Alcantara JMA, Sanchez-Delgado G, Martinez-Tellez B, Labayen I, Ruiz JR. Impact of cow’s milk intake on exercise performance and recovery of muscle function: a systematic review. J Int Soc Sports Nutr. 2019 Dec;16(1):22.
3. Amiri M, Ghiasvand R, Kaviani M, Forbes SC, Salehi-Abargouei A. Chocolate milk for recovery from exercise: a systematic review and meta-analysis of controlled clinical trials. Eur J Clin Nutr. 2019 Jun;73(6):835–49.
4. Newell M, Wallis G, Hunter A, Tipton K, Galloway S. Metabolic Responses to Carbohydrate Ingestion during Exercise: Associations between Carbohydrate Dose and Endurance Performance. Nutrients. 2018 Jan 3;10(1):37.
5. O’Hara JP, Carroll S, Cooke CB, Morrison DJ, Preston T, King RFGJ. Preexercise Galactose and Glucose Ingestion on Fuel Use during Exercise. Med Sci Sports Exerc. 2012 Oct;44(10):1958–67.

## Poster Presentations

### P1 – Cluster-set resistance training based on loss of velocity on exercise performance in young adults

#### José E. Bimbela-Villalobos^1^, Luis Mario Gómez-Miranda^1^, Melinna Ortiz-Ortiz^1^, Jorge A. Aburto-Corona^1^, Roberto Espinoza-Gutiérrez^1^, Juan J. Calleja-Núñez^1^, Daniel Rojas-Valverde^2,3^, Juan D. Ascuntar-Viteri^4^, Diego A. Bonilla^1,5^

##### ^1^Facultad de Deportes, Universidad Autónoma de Baja California, Tijuana, México; ^2^Núcleo de Estudios para el Alto Rendimiento y la Salud (CIDISAD-NARS), Escuela Ciencias del Movimiento Humano y Calidad de Vida (CIEMHCAVI), Universidad Nacional, Heredia, Costa Rica; ^3^Clínica de Lesiones Deportivas (Rehab&Readapt), Escuela Ciencias del Movimiento Humano y Calidad de Vida (CIEMHCAVI), Universidad Nacional, Heredia, Costa Rica; ^4^Research Division, Dynamical Business & Science Society—DBSS International SAS, Bogota, Colombia; ^5^Research Group in Physical Activity, Sports and Health Sciences (GICAFS), Universidad de Córdoba, Monteria 230002, Colombia

###### **Correspondence:** Luis Mario Gómez-Miranda (lgomez8@uabc.edu.mx)

*BMC Proceedings 2024*, **18(Suppl 4)**P1

**Introduction:** This study compared two cluster-set resistance training (CL-RT) protocols controlled by velocity loss (VL) on performance in squat (SQ), bench press (BP), and conventional deadlift (DL) exercises in recreationally trained men. **Methods:** This single-arm crossover randomized clinical trial involved 12 resistance-trained men (1+ years of training experience), aged 18 to 35 years. Three separate sessions were conducted with a 48 to 96-hour rest. The first session included anthropometry measurements, one-repetition maximum (1-RM) tests and mean propulsive velocity (MPV) measurements using a linear position transducer to establish individual load-velocity (L-V) profiles for the exercises (1,2). Finally, the order of the CL-RT20 and CL-RT40 experimental sessions was randomized (3). Both experimental sessions began with a 10-minute treadmill warm-up and identification of 80% 1-RM based on the L-V profile to set the working sets. Each session comprised three sets of as many repetitions as possible until a 10% VL, followed by a rest interval of either 20 or 40 seconds, and then continued with as many repetitions as possible until a 10% VL again. The rest between exercises was five minutes. The tempo for all sessions was 2-0-X-0 in SQ, 2-1-X-0 in BP, and 2-2-X-1 in DL. **Results:** For both SQ and BP, total repetitions and total volume were significantly higher in CL-RT40 compared to CL-RT20 (*P*=0.001); however, no significant differences were found in DL. **Conclusions:** VL seems to be useful in configuring the CL-RT program with heavy loads to enhance manifestations of muscle strength. Probably, due to the longer rest interval within the set, the CL-RT40 configuration yields better results in SQ and BP performance, although it does not seem superior in DL when compared to CL-RT20.

**References**

1. Jukic I, Castilla AP, Ramos AG, Van Hooren B, McGuigan MR, Helms ER. The Acute and Chronic Effects of Implementing Velocity Loss Thresholds During Resistance Training: A Systematic Review, Meta-Analysis, and Critical Evaluation of the Literature. *Sports Med.* 2023; 53(1): 177-214.
2. Baena-Marín M, Rojas-Jaramillo A, González-Santamaría J, Rodríguez-Rosell D, Petro JL, Kreider RB, Bonilla DA. Velocity-Based Resistance Training on 1-RM, Jump and Sprint Performance: A Systematic Review of Clinical Trials. *Sports (Basel)*. 2022; 10(1): 8.
3. Vargas-Molina S, Romance R, Schoenfeld BJ, Garcia M, Petro JL, Bonilla DA, Kreider RB, Martin-Rivera F, Benitez-Porres J. Effects of cluster training on body composition and strength in resistance-trained men. *Isokinet. Exerc. Sci*. 2020; 28(4): 391-399.

### P2 - Anthropometric profiles and body composition in CrossFit® athletes

#### Erleny Rincón-Quintero, Stephanny Orozco-López, Alejandra Velásquez, Santiago Valencia-Rivera, Brigitt Berdugo-Alomias

##### Institución Universitaria Escuela Nacional del Deporte, Cali, Valle del Cauca, Colombia

###### **Correspondence:** Brigitt Berdugo-Alomias (brigitt.berdugo@endeporte.edu.co)

*BMC Proceedings 2024*, **18(Suppl 4)**P2

**Introduction:** CrossTraining, a high-intensity exercise training, has gained popularity in recent years. This study aimed to describe the anthropometric profile and body composition of athletes based on their gender and category participating in the CrossFit® WolfGames 2022 competition in Cali (Valle del Cauca, Colombia). **Methods:** A total of 100 adult CrossTrainers (62M and 38F) with at least one year of CrossFit® experience enrolled in the WolfGames 2022 participated in this cross-sectional study. Participants were classified into different categories (beginners, intermediates, advanced, and elite). The restricted profile of the International Society for the Advancement of Kinanthropometry (ISAK) was evaluated by certified L1 and L2 anthropometrists (1). Measurements were taken over two days, coinciding with the event’s duration. The percentage of body fat (%BF) was estimated using the Yuhasz equation (2). Additionally, the sum of six skinfold thicknesses (ΣSS), the active substance index (IAKS), the Cormic index (CI), the proportionality index, the relative arm span, and the Heath & Carter somatotype were calculated (3). **Results:** Women had higher values of individual skinfolds (particularly thigh and medial calf), and significant differences were observed on ΣSS, %BF, and IAKS when beginner and intermediate females were compared. Conversely, no significant differences were found in BMI and IAKS in male athletes from beginner and intermediate categories, although ΣSS was higher in beginners as expected. Predominance of brachioskeletal and high CI were observed in both categories. Advanced and elite CrossTrainers showed lower values of %BF and CI, although ΣSS was higher compared to both beginners and intermediates. Mesomorphy was predominant among all male categories. **Conclusions:** The anthropometric profiles of female and male CrossTrainers from different categories were described. These results can serve as a reference for establishing monitoring parameters and nutritional guidelines; however, more research is needed to evaluate the relationships of these indicators with physical performance.

**References**

1. Esparza-Ros F, Vaquero-Cristóbal R, Marfell-Jones M. International Standards for Anthropometric Assessment. The International Society for the Advancement of Kinanthropometry; Murcia, Spain: 2019.
2. Yuhasz MS. The effects of sports training on body fat in man with predictions of optimal body weight. PhD Thesis, University of Illinois at Urbana-Champaign, 1962.
3. Heath BH, Carter JE. A modified somatotype method. *Am J Phys Anthropol*. 1967; 27(1): 57-74.

### P3 - Effect of glycerol plus sodium on hydration status, time to exhaustion, and cardiorespiratory fitness in elite Colombian cyclists: protocol of a randomized clinical trial

#### Laura G. Ortegón-Martínez^1^, Erik Bejarano^2^, Camilo E. Povea-Combariza^2^, Ligia S. Guerrero-Orjuela^3^, Astrid E. Quinchía^2^

##### ^1^Maestría en Fisioterapia del Deporte y la Actividad Física, Universidad Nacional de Colombia, Bogotá, Colombia; ^2^Centro de Ciencias del Deporte, Ministerio del Deporte, Bogotá, Colombia; ^3^Departamento de Nutrición Humana, Universidad Nacional de Colombia, Bogotá, Colombia

###### **Correspondence:** Laura G. Ortegón-Martínez (lgortegonm@unal.edu.co)

*BMC Proceedings 2024*, **18(Suppl 4)**P3

**Introduction:** Although glycerol (GLY) has been proposed as a hyperhydrating agent with the potential to improve cardiorespiratory fitness (CRF), there is still some hesitancy about its use, as it was only removed from the list of prohibited substances by the World Anti-Doping Agency in 2018. Considering the lack of research combining GLY with other substances, this protocol describes a clinical trial aiming to evaluate the effects of GLY plus sodium (Na^+^) on hydration status and physical performance in elite Colombian cyclists. **Methods:** This randomized, double-blind, crossover clinical trial will involve 20 Colombian male professional cyclists aged 18 to 30 years, belonging to the Colombian Cycling Federation in the classic race modality, with a minimum of 2 years of high-performance sports life and training for a minimum of 15 hours per week. The total study duration will be 6 weeks, during which three drinks (GLY + Na^+^, GLY, and water) will be administered with a one-week washout period between them. In the laboratory, a CRF test on a cycle ergometer for 120 minutes at 65% of VO_2max_ and an incremental resistance test will be conducted. In the field, a 44 km ride at 70% of VO_2max_ will be completed at temperatures of 25°C and altitudes below 400 m above sea level. Primary outcomes include excreted urine volume, urine specific gravity, urine osmolality, time to exhaustion, and functional threshold power. **Expected results:** The alternative hypothesis addressed in the study is that the GLY + Na^+^ drink is an effective ergogenic aid for endurance cyclists as it improves hydration status and physical performance under increased physical load without causing significant gastrointestinal symptoms, as compared to other drinks.

**References**

1. Goulet ED, Aubertin-Leheudre M, Plante GE, Dionne IJ. A meta-analysis of the effects of glycerol-induced hyperhydration on fluid retention and endurance performance. *Int J Sport Nutr Exerc Metab*. 2007; 17(4): 391-410.
2. van Rosendal SP, Osborne MA, Fassett RG, Coombes JS. Guidelines for glycerol use in hyperhydration and rehydration associated with exercise. *Sports Med*. 2010; 40(2): 113-29.
3. Goulet EDB, De La Flore A, Savoie FA, Gosselin J. Salt + Glycerol-Induced Hyperhydration Enhances Fluid Retention More Than Salt- or Glycerol-Induced Hyperhydration. *Int J Sport Nutr Exerc Metab*. 2018; 28(3): 246-52.

### P4 - Relationship between phase angle, body composition parameters and handgrip strength

#### Leonardo Rodríguez-Perdomo, July P. Moreno-Alvarado, Samuel Hernández-Almanza

##### Centro de Formación en Actividad Física y Cultura, Servicio Nacional de Aprendizaje – SENA, Bogotá, Colombia

###### **Correspondence:** Leonardo Rodríguez-Perdomo (leorodriguezp@sena.edu.co)

*BMC Proceedings 2024*, **18(Suppl 4)**P4

**Introduction:** The phase angle (PhA) has been established as an indicator of nutritional status in adult and infant populations [1], as well as in different levels of physical activity [2]. This study aimed to determine the relationship between PhA, estimates of body composition (BC), and handgrip strength (HGS) in both hands in a population from Palmira (Valle del Cauca), aiming to establish cutoff values for this Colombian region. **Methods:** After the call to participate, 107 technology degree students from Palmira (63F, 44M) signed the informed consent and participated in this cross-sectional study. Anthropometric variables were taken according to the standards of the International Society for the Advancement of Kinanthropometry (ISAK). For Bioelectrical Impedance Analysis (BIA), a multifrequency octopolar device (SECA® mBCA 525) was used following recommended procedures. Finally, HGS was measured to the nearest 100 g using an analogous device (Smedley III T-18A) considering the protocol of the American Society of Hand Therapists (ASHT). **Results:** HGS and most anthropometric variables, except for waist girth, visceral and total fat, exhibited a normal distribution (*P*>0.05). Pearson tests revealed low-to-medium correlations between PhA and body mass (*r*=0.23), stature (*r*=0.51), BMI (*r*=-0.11), body fat (*r*=-0.46), visceral fat (*r*=-0.27), waist girth (*r*=-0.07), and intracellular water (*r*=-0.30). However, PhA showed a positive medium-to-high correlation with right HGS (*r*=0.58) and left HGS (*r*=0.62). Additionally, high-to-very high correlations were found between PhA and lean mass (*r*=0.69), muscle mass (MM, *r*=0.74), trunk MM (*r*=0.75), right-leg MM (*r*=0.68), left-leg MM (*r*=0.70), right-arm MM (*r*=0.74), and left-arm MM (*r*=0.73). Regression models were generated for PhA from the other variables. **Conclusions:** This study provides evidence showing a strong relationship between PhA and HGS variables, total MM, and lean mass. Further research is needed to understand the relationships between PhA and cardiometabolic risk variables.

**Funding**

This research received funding from CFAFC – SENNOVA.

**References**

1. Aldobali M, Pal K. Bioelectrical impedance analysis for evaluation of body composition: A review. *IEEE*; In International Congress of Advanced Technology and Engineering (ICOTEN) 2021: 1-10.
2. Yamada Y, Yoshida T, Murakami H, Kawakami R, Gando Y, Ohno H, Tanisawa K, Konishi K, Julien T, Kondo E, Nakagata T, Nanri H, Miyachi M. Phase angle obtained via bioelectrical impedance analysis and objectively measured physical activity or exercise habits. Sci Rep. 2022; 12(1): 17274.

### P5 - Effect of physical and mental effort on frontal lobe beta electric activity in young university students

#### Nicolás Córdoba-Jiménez, Jesús Astolfo Romero-García, Felipe Garavito-Peña

##### Grupo de Investigación GICAEDS, Facultad de Cultura Física, Deporte y Recreación, Universidad Santo Tomás, Bogotá, Colombia

###### **Correspondence:** Nicolás Córdoba-Jiménez (nicolas.cordoba@usantotomas.edu.co)

*BMC Proceedings 2024*, **18(Suppl 4)**P5

**Introduction:** While the neurotrophic effect of exercise on the brain is evident, it remains unclear how different exercise activities modulate electrical activation of the cerebral cortex. In this study, we assessed the effect of physical and mental effort on beta electrical activity in the frontal lobe in young university students. **Methods:** Ten male university students (19.9 [1.8] years; 62.28 [5.52] kg; 171.1 [3.51] cm) participated in this non-randomized feasibility pilot study. The Emotiv Epoc® wireless brain-interface device was used for electroencephalographic (EEG) recording, following the recommendations of the International Federation of Clinical Neurophysiology [1]. The intervention protocol was randomized for each day as follows: Protocol 1, Resting EEG + Mental Effort (Wais and Stroop tests); Protocol 2, Strict Cardiovascular Effort; Protocol 3, Strict Cardiovascular Effort + Mental Effort; and Protocol 4, Coordination Effort. Resting values of heart rate, heart rate variability, brain activity, and ergospirometry were recorded as secondary outcomes for ten minutes in a seated position. **Results:** Statistically significant changes were observed for the different evaluated protocols. In terms of power spectrum, Protocol 2 (13.54 [6.23] uV, *P*<0.0001) and Protocol 3 (15.11 [4.61] uV, *P*<0.0001) exhibited higher power compared to rest and other protocols. In the frequency spectrum (Hz), processing speed work (Stroop) at rest (18.45 [4.25] Hz, *P*<0.002) was higher compared to Protocols 2 and 4. **Conclusions:** EEG electrical activity in power spectra increases above rest levels when adding physical, coordinative, and mental effort loads. At the frequency range of 13-30 Hz, tasks involving higher concentration and cortical activation, such as processing speed (Stroop), tend to have a longer wave cycle compared to strict cardiovascular and coordination work in acute measurements. The results obtained may provide a baseline for understanding the brain’s energy expenditure during different types of physical activity in future research.

**Funding**

This research received funding from ‘Fondo de Desarrollo de la Investigación’ FODEIN 2023 at Universidad Santo Tomás (Bogotá, Colombia). Also, we acknoledge the access to the call ‘Joven Investigador’ from FODEIN Multicampus 2022.

**References**

1. Lopes da Silva F. EEG and MEG: relevance to neuroscience. *Neuron*. 2013; 80(5): 1112-28
2. Voelcker-Rehage C, Godde B, Staudinger UM. Physical and motor fitness are both related to cognition in old age. *Eur J Neurosci*. 2010; 31(1): 167-176.

## Abstracts

### A1 - Fractal dimension and potential for body composition assessment: a pilot study

#### Luis F. Bedoya-Bedoya^1^, Katherine Franco-Hoyos^1^, Alejandra Agudelo-Martínez^1^, Diego A. Bonilla^1,2,3^

##### ^1^Grupo de Investigación NUTRAL, Facultad de Ciencias de Nutrición y Alimentos, Universidad CES, Medellín, Colombia; ^2^Research Division, Dynamical Business & Science Society – DBSS International SAS, Bogotá, Colombia; ^3^Red Iberoamericana de Investigadores en Antropometría Aplicada – RIBA2, España

###### **Correspondence:** Diego A. Bonilla (dabonilla@dbss.pro)

*BMC Proceedings 2024*, **18(Suppl 4)**A1

**Introduction:** Fractal dimension (FD) is a mathematical concept that quantifies the complexity of a geometric shape by measuring its degree of self-similarity at different scales [1,2]. Given the lack of knowledge and standardization in digital anthropometry, this study aimed to explore the potential of FD to estimate body composition through the analysis of silhouettes and dual-energy X-ray absorptiometry (DXA) data as a reference. **Methods:** We recruited 21 healthy participants (both women and men) aged 18 to 30 years. The pilot study took place at the Center for Advanced Studies in Nutrition and Food (CESNUTRAL) at CES University in Medellin, Colombia. Digital photographs were taken at distances of 1 and 2 meters in spaces with artificial lighting and white background using the Fit Your Outfit app to ensure proper camera framing. The camera was positioned at the participants’ *omphalion* height, and the arms were maintained between 30° and 45°. Body composition was assessed using a Lunar Prodigy™ unit (General Electric Healthcare, USA). FD analysis was performed using the Fractal Dimension Estimator software. **Results:** Progress was made in standardizing photographic records in three ISAK-recommended positions: a) anthropometric position, b) anatomical position, c) lateral anthropometric position. Preliminary analyses revealed no correlations between FD values of silhouettes in anthropometric and anatomical positions at 1 and 2 meters with total fat mass or gynoid fat mass values obtained by DXA. However, a positive correlation trend was identified between FD and android fat mass. **Conclusions:** This preliminary data suggest that FD does not correlate with total fat mass or gynoid fat mass values in any photographic records. However, the behavior of FD regarding android fat mass requires further investigation. Data analysis will continue by modifying FD estimation parameters and comparing against other metrics derived from DXA.

**References**

1. Ziukelis ET, Mak E, Dounavi ME, Su L, J TOB. Fractal dimension of the brain in neurodegenerative disease and dementia: A systematic review. *Ageing Res Rev*. 2022;79:101651.
2. Beretta-Piccoli M, D’Antona G, Barbero M, Fisher B, Dieli-Conwright CM, Clijsen R, et al. Evaluation of central and peripheral fatigue in the quadriceps using fractal dimension and conduction velocity in young females. *PLoS One.* 2015; 10(4): e0123921.

### A2 - Fluid replenishment and sweating rate in soccer players: a comparison between starters and substitutes

#### Jacobo Martínez^1,2^, Ricardo C. Rivera-Cortez^3^, Hugo D. Rodríguez-Jiménez^3^, Mónica G. Reyes-Monroy^3^, Hernani Rea-Páez^3^, María A. Negrete-Castellano^3^, Luz M. Frias-Vázquez^3^, Herica Arias-Mojarro^3^, Diego A. Bonilla^1,2^

##### ^1^Research Division, Dynamical Business & Science Society – DBSS International SAS, Bogotá, Colombia; ^2^Grupo de investigación NUTRAL, Facultad Ciencias de la Nutrición y los Alimentos, Universidad CES, Medellín, Colombia; ^3^Licenciatura en Nutrición, Unidad Académica de Salud Integral, Universidad Autónoma de Nayarit, Tepic, México

###### **Correspondence:** Herica Arias-Mojarro (herica.arias@uan.edu.mx)

*BMC Proceedings 2024*, **18(Suppl 4)**A2

**Introduction:** Isotonic drinks with carbohydrates and electrolytes have been reported to prevent fatigue caused by fluid and energy loss [1]. However, excess glucose in these drinks could impact fluid replenishment [2]. This study aimed to assess differences between two hydration plans in young soccer players, starters receiving a commercial isotonic drink and substitutes receiving regular water. **Methods:** A total of 30 players from the 2003 and 2004 categories of the Santos Tepic Sub15 Football Academy in the Jalisco State Football Association (Mexico) were evaluated. Anthropometric assessments were conducted following ISAK guidelines. Body mass data were collected before and after training, and sweating rate (L·h^-1^) was estimated according to Dunford (2006) [3]. Two types of drinks were used for hydration control: an isotonic drink (Gatorade®) for starters and natural water for substitutes, provided in quantities of 250 ml every 20 minutes. **Results:** The robust analysis of trimmed means and winsorized standard deviations revealed no significant differences on hydration status (percentage of post-exercise body mass loss) or sweating rate when comparing starters (who consumed the commercial drink) with substitutes (who only had water). **Conclusions:** In the context of this study, it seems that Sub15 substitute soccer players may not need to consume commercial isotonic drinks as long as appropriate amounts of water are administered periodically during physical exertion.

**Funding**

This research was funded by the Unidad Académica de Salud Integral at Universidad Autónoma de Nayarit (NOM-004-SSA3-2012).

**References**

1. Kenefick RW. Drinking Strategies: Planned Drinking Versus Drinking to Thirst. *Sports Med.* 2018; 48(Suppl 1): 31-7.
2. O’Connell SM, Woodman RJ, Brown IL, Vincent DJ, Binder HJ, Ramakrishna BS, et al. Comparison of a sports-hydration drink containing high amylose starch with usual hydration practice in Australian rules footballers during intense summer training. *J Int Soc Sports Nutr.* 2018; 15(1): 46.
3. Dunford M. Sports Nutrition: a practice manual for professionals. 4ta Ed. USA: American Dietetic Association: 2006.

### A3 - Hydration status and fluid replacement strategies in young soccer players: an integrative systematic review

#### Diego G. Morales-Rosillo^1^, Georgina Vargas-Covarrubias^2^, Niza M. Estrada Cortés^2^, Luis M. Gómez-Miranda^3^, Herica Arias-Mojarro^2^, Katherine Franco^4^, Juan Diego Quintero^1^, Jana Kočí^1,5^, Diego A. Bonilla^1,4^

##### ^1^Research Division, Dynamical Business & Science Society – DBSS International SAS, Bogotá, Colombia; ^2^Licenciatura en Nutrición, Unidad Académica de Salud Integral, Universidad Autónoma de Nayarit, Tepic, México; ^3^Facultad de Deportes, Universidad Autónoma de Baja California, Tijuana, México; ^4^Grupo de investigación NUTRAL, Facultad Ciencias de la Nutrición y los Alimentos, Universidad CES, Medellín, Colombia; ^5^Department of Education, Faculty of Education, Charles University, Prague, Czech Republic

###### **Correspondence:** Diego A. Bonilla (dabonilla@dbss.pro)

*BMC Proceedings 2024*, **18(Suppl 4)**A3

**Introduction:** It is essential to individually monitor the loss of fluids and electrolytes for each athlete [1]. This integrative systematic review compiles and analyzes available evidence to date to describe hydration status and fluid replacement strategies in young soccer players, providing practical recommendations for sports nutritionists and dietitians. **Methods:** A search for randomized controlled trials, case studies, narrative reviews, systematic reviews, and meta-analyses published between 2012 and 2023, analyzing fluid replacement strategies in young soccer players, was conducted. The search was performed on Pubmed/MEDLINE, Science Direct, Google Scholar, and PEDro using Boolean algorithms. Risk of bias assessment for randomized controlled trials, non-randomized trials, and reviews was done using RoB 2.0, Robins, and AMSTAR 2.0 tools, respectively. Selected studies were analyzed for: i) demographic aspects of the study population; ii) training protocol; iii) sweating rate; iv) pre-post body mass; v) electrolyte loss; and vi) fluid replacement strategies. **Results:** Seventeen studies, with a total of 1153 participants (1013 males, 140 females), conducted mainly in USA, Brazil, Belgium, Turkey, Spain, Canada, South Africa, and UK were retrieved. Specific gravity of urine and percentage loss of body mass are common methods for assessing hydration status. A high prevalence of low hydration [24%-90%] (>1.020 USG) and low fluid intake in cool climates were identified. Finally, *ad libitum* strategy appears not to prevent greater dehydration in young soccer players. **Conclusions:** Our findings describe a high prevalence of dehydration before sports activity in young soccer players. However, we highlight the lack of studies on female youth soccer players. This work provides a set of practical recommendations for fluid replacement that may have a positive impact on the hydration status of the infant-juvenile population.

**Reference**

1. Shirreffs SM, Sawka MN, Stone M. Water and electrolyte needs for football training and match-play. *J. Sports Sci*. 2006; 24(07): 699-707.

### A4 - Cardiorespiratory fitness profiles of athletes residing at high altitude through machine learning

#### Miller E. Vargas-Santiago^1,2^, Juan Zuñiga^1,2^, Camilo A. Rincón-Yepes^1,2^, Carlos E. Montenegro-Marín^3^, Richard B. Kreider^4^, Jorge L. Petro^1,5^, Diego A. Bonilla^1,5^

##### ^1^Research Division, Dynamical Business & Science Society – DBSS International SAS, Bogotá, Colombia; ^2^CenRED - Centro de Rehabilitación, Entrenamiento y Desempeño Deportivo, Bogotá, Colombia; ^3^Facultad de Ingeniería, Universidad Distrital Francisco José de Caldas, Bogotá, Colombia; ^4^Exercise & Sport Nutrition Laboratory, Human Clinical Research Facility, Texas A&M University, College Station, USA; ^5^Research Group in Physical Activity, Sports and Health Sciences (GICAFS), Universidad de Córdoba, Montería, Colombia

###### **Correspondence:** Diego A. Bonilla (dabonilla@dbss.pro)

*BMC Proceedings 2024*, **18(Suppl 4)**A4

**Introduction**: Evaluating maximum oxygen consumption (VO_2max_) reveals the level of physiological adaptation to physical exercise, influencing athletic performance. This study aims to profile the cardiorespiratory fitness of athletes trained at high altitude (2600 - 3700 meters above sea level) using unsupervised machine learning. **Methods**: A cross-sectional study collected basic anthropometric and cardiorespiratory fitness-related data following Strengthening the Reporting of Observational Studies in Epidemiology (STROBE) guidelines [1]. Cardiopulmonary exercise testing was conducted using the BTL Cardiopoint Ergo Pro E600 system (BTL Medical Technologies, CZ). After comparing different clustering algorithms (k-means, k-medoids, hierarchical, and CLARA), a classification analysis was performed. **Results**: Out of the initial 90 subjects, 26 were excluded due to incomplete data. Therefore, a total of 64 subjects (21F, 43M) were analyzed. The sample included four professional soccer players (Colombian women’s national team) and one elite long-distance runner, with the rest being recreationally endurance athletes. Internal validation suggested hierarchical clustering with k = 2, using the Ward minimum variance criterion. Two significantly different profiles emerged: i) sex-independent, older, slower, with VO_2max_ values of 49.4 (8.4) ml/kg/min [46.28 – 52.16] (Cluster 1, *n* = 34); and ii) younger, taller, faster subjects, mainly men (93%), with excellent VO_2max_ values of 64.7 (8.0) ml/kg/min [61.68 – 67.73] (Cluster 2, *n* = 30). The data is presented as mean (standard deviation) [95% confidence interval]. **Conclusions**: Two profiles of athletes trained at high altitude were identified. Cluster 2 subjects exhibited higher cardiorespiratory capacity compared to Cluster 1. Beyond aiding training planning, these results contribute methodological and physiological insights into individual responses to high-altitude training.

**Reference**

1. Vandenbroucke JP, von Elm E, Altman DG, Gøtzsche PC, Mulrow CD, Pocock SJ, Poole C, Schlesselman JJ, Egger M; STROBE Initiative. Strengthening the Reporting of Observational Studies in Epidemiology (STROBE): explanation and elaboration. *PLoS Med.* 2007; 4(10): e297.

### A5 - Identifying components of allostatic load for monitoring exercise stress-induced adaptations in elite basketball players

#### Andrés García^1^, Juan Manuel-Lopez^2^, Andrés Rojas-Jaramillo^1,2^, Gustavo Humeres^1,3^, Jhonatan González-Santamaria^1,4,5^, Jeffrey R. Stout^6^, Alejandra Agudelo-Martínez^7^, Diego A. Bonilla^1,7^

##### ^1^Research Division, Dynamical Business & Science Society – DBSS International SAS, Bogotá, Colombia; ^2^Grupo de Investigación GRICAFDE, Instituto Universitario de Educación Física y Deportes, Universidad de Antioquia, Medellín, Colombia; ^3^Instituto de Ciencias de Rehabilitación y el Movimiento (ICRM), Universidad Nacional de San Martín, Buenos Aires, Argentina; ^4^Grupo de Investigación ZIPATEFI, Fundación Universitaria del Área Andina, Pereira, Colombia; ^5^Grupo Investigación y Desarrollo en Cultura de la Salud, Universidad Tecnológica de Pereira, Pereira, Colombia; ^6^Physiology of Work and Exercise Response (POWER) Laboratory, University of Central Florida, Orlando, USA; ^7^Grupo de Investigación NUTRAL, Facultad de Ciencias de Nutrición y Alimentos, Universidad CES, Medellín, Colombia

###### **Correspondence:** Rojas-Jaramillo (arojasj@unal.edu.co); Diego A. Bonilla (dabonilla@dbss.pro)

*BMC Proceedings 2024*, **18(Suppl 4)**A5

**Introduction:** The allostatic load (AL) index is derived from a set of molecular and physiological biomarkers that indicate sub-clinical or clinical deviations [1], providing insights into the adaptation process and enabling the anticipation of stress-related requirements. This integrative and allostatic perspective has facilitated the identification of potential new biomarkers [2]. This pilot study aimed to evaluate the relationships among various molecular, physical, and psychometric biomarkers with the goal of constructing a valid AL index for professional team sport athletes. **Methods:** Twelve Colombian elite male basketball players (18.3 [0.9] years; 77.2 [5.7] kg; 185 [9.0] cm) from the Antioquia team underwent an identical training program, with two microcycles dedicated to load and fatigue estimation (18 days in total). Counter-movement jump (CMJ), creatine kinase (CK), blood urea levels, the Foster index (calculated as the product of the Borg-10 subjective perception of effort scale and training duration in minutes), and perceived general well-being (evaluated for sleep, stress, fatigue, delayed onset muscle soreness, and mood) were assessed at the end of each session. **Results:** The findings revealed a statistically significant correlation between molecular biomarkers and the quality of sleep (*P*<0.05). Specifically, CK exhibited a significant negative correlation with perceived sleep quality, indicating that higher CK levels corresponded to lower perceived sleep quality. Blood urea levels also displayed a significant correlation with the perception of sleep quality and delayed onset muscle soreness, while CMJ showed a significant correlation with subjective perception of effort. Finally, the Foster Index demonstrated associations with pre-training CMJ, post-training CMJ, and sleep quality. **Conclusions:** This study highlights significant relationships between molecular and physical biomarkers with well-being variables, particularly sleep quality, muscle pain, and the cumulative score. Additional validation research is warranted to develop an AL index that incorporates psychometric variables in professional team sport athletes.

**References**

1. Bobba-Alves N, Juster RP, Picard M. The energetic cost of allostasis and allostatic load. *Psychoneuroendocrinology*. 2022; 146: 105951.
2. Bonilla DA, Moreno Y, Petro JL, Forero DA, Vargas-Molina S, Odriozola-Martínez A, Orozco CA, Stout JR, Rawson ES, Kreider RB. A Bioinformatics-Assisted Review on Iron Metabolism and Immune System to Identify Potential Biomarkers of Exercise Stress-Induced Immunosuppression. *Biomedicines*. 2022; 10(3): 724.

### A6 - Effects of an anterior cruciate ligament rehabilitation program based on cross-education on morphological and functional changes in lower limbs

#### Jorge M. Vélez-Gutiérrez^1,2^

##### ^1^ARTHROS Centro de Fisioterapia y Ejercicio, Medellín 050012, Colombia; ^2^Research Division, Dynamical Business & Science Society – DBSS International SAS, Bogotá, Colombia

###### **Correspondence:** Jorge M. Vélez-Gutiérrez (arthros.gerencia@gmail.com)

*BMC Proceedings 2024*, **18(Suppl 4)**A6

**Introduction:** This study aimed to assess the effectiveness of a cross-education-based rehabilitation program (CE-RP) in mitigating neuromuscular changes post anterior cruciate ligament (ACL) injury/reconstruction [1]. It also evaluated its impact on pre- and post-surgery quadriceps and hamstring cross-sectional areas and compared effects on muscle activation and strength levels with conventional rehabilitation [2-3]. **Methods:** Seven male participants were randomly assigned to the CE-RP (*n* = 4) or control group (CG, *n* = 3) and underwent an 8-week rehabilitation program accordingly. Quadriceps and hamstrings’ cross-sectional areas (CSA) were assessed using magnetic resonance imaging (MRI). Maximal voluntary isometric contraction (MVIC) and rate of force development (RFD) of the quadriceps during 90° knee extension were recorded with a dynamometer. Hamstrings’ MVIC and RFD were assessed using the 90/20 isometric strength test. Jump height and relative strength index (RSI) were measured during the single-leg countermovement jump (SL-CMJ) test. Muscle activity was evaluated through surface electromyography (EMG). All measurements were taken pre- and post-intervention. **Results:** The CE-RP demonstrated increased MVIC and RFD in the quadriceps, whereas only 66.6% of the CG showed improvements. Additionally, 50% of the CE-RP increased mean and peak force in the hamstrings, compared to 66.6% and 100% in the CG, respectively. For hamstring RFD, 25% of the CE-RP increased while 66.6% of the CG did. Furthermore, 50% of the CE-RP increased jump height and RSI, compared to 100% and 33% in the CG, respectively. No significant differences were observed when comparing changes of the CE-RP versus CG. **Conclusions:** A CE-RP program was not superior to conventional rehabilitation program to preserve muscle mass based on MRI cross-sectional area. Although cross-education led to superior strength and muscle activation gains compared to conventional rehabilitation, no significant differences or interactions were found. More research is needed considering the limited sample study size.

**References**

1. Harput G, Ulusoy B, Yildiz TI, Demirci S, Eraslan L, Turhan E, Tunay VB. Cross-education improves quadriceps strength recovery after ACL reconstruction: a randomized controlled trial. *Knee Surg Sports Traumatol Arthrosc*. 2019; 27(1): 68-75.
2. Barss TS, Pearcey GE, Zehr EP. Cross-education of strength and skill: an old idea with applications in the aging nervous system. *Yale J Biol Med*. 2016; 89(1): 81-86.
3. Manca A, Hortobágyi T, Carroll TJ, Enoka RM, Farthing JP, Gandevia SC, Kidgell DJ, Taylor JL, Deriu F. Contralateral Effects of Unilateral Strength and Skill Training: Modified Delphi Consensus to Establish Key Aspects of Cross-Education. *Sports Med*. 2021; 51(1): 11-20.

### A7 - Associations of serum cystatin C and creatinine levels with body composition, nutrition, and pharmacological practices in Mexican competitive bodybuilders: a cross-sectional study

#### Kevin E. Villa^1^, Clara B. Mercado^1^, Alin J. Palacios^1^, Melinna Ortiz^2^, Rolando Posada-Florez^3^, Luis M. Gómez-Miranda^4^, Alejandra Agudelo-Martínez^5^, Diego A. Bonilla^3,5^

##### ^1^Licenciatura en Nutrición, Facultad de Medicina, Universidad de Colima, Colima, México; ^2^Facultad de Medicina y Psicología, Universidad Autónoma de Baja California, Tijuana, México; ^3^Research Division, Dynamical Business & Science Society – DBSS International SAS, Bogotá, Colombia; ^4^Facultad de Deportes, Universidad Autónoma de Baja California, Tijuana, México; ^5^Grupo de Investigación NUTRAL, Facultad de Ciencias de Nutrición y Alimentos, Universidad CES, Medellín, Colombia

###### **Correspondence:** Kevin E. Villa (kvilla3@ucol.mx)

*BMC Proceedings 2024*, **18(Suppl 4)**A7

**Introduction:** Renal failure is a prevalent risk among bodybuilders using anabolic-androgenic steroids (AAS) or other performance- and image-enhancing drugs (PIEDs) [1,2]. This study examined the relationship between renal biomarkers and nutritional/pharmacological practices in Mexican bodybuilding competitors. **Methods:** A cross-sectional study included fifteen PIEDs-using bodybuilders (aged 18 to 50) planning to compete at the ‘*Asociación de Fisicoculturismo y Fitness del Estado de Colima*’ (AFFEC, Mexico). Participants were categorized as off-season (OFF, *n* = 7, 76.98 [11.0] kg) or Peak Week (PW, *n* = 8, 82.02 [13.4] kg), considering an 8-week threshold before competition. Blood samples were analyzed for Cystatin C (CysC, immunoturbidimetry) and creatinine (spectrophotometry) concentrations, enabling glomerular filtration rate (GFR) estimation using various equations. Dietary intake was assessed via 24-hour recall, a questionnaire covered PIEDs use, and adiposity and muscularity were measured using sum of skinfolds (ΣSS) and corrected girths (ΣCG), respectively. **Results:** Estimated GFR varied across equations (20-60% participants showed decreased renal function), with CysC-based equations exhibiting less dispersion. Although not statistically significant, higher [CysC] with a large effect size were found in PW compared to OFF (*d*=0.79). Dietary protein and lipids, and AAS use were higher in PW (*d*=0.80, 0.73, and 0.84, respectively). A non-significant positive correlation was found between AAS and [CysC] at OFF (r=0.64) but was negative at PW (r=-0.54). There was a non-significant moderate correlation between calf ΣCG and [CysC] at PW (r=0.43), with a significant high correlation at OFF (r=0.86, 95%CI 0.20, 0.97). As expected, a significant positive correlation was found between AAS and ΣCG (r=0.53, 95%CI 0.03, 0.82), specially at OFF (r=0.78 (CI 0.07, 0.97) while non-significant moderate correlation at PW (r=0.49). Finally, a significant negative moderate correlation was found between AAS use and ΣSS in all subjects (r=-0.57, CI -0.84, -0.09), in OFF (r=-0.48), and in PW (r=-0.50). **Conclusions**: This study provide insights into the complex interplay between nutritional/pharmacological choices, renal function, and body composition in bodybuilding competitors.

**References**

1. Pope HG Jr, Wood RI, Rogol A, Nyberg F, Bowers L, Bhasin S. Adverse health consequences of performance-enhancing drugs: an Endocrine Society scientific statement. *Endocr Rev.* 2014; 35(3): 341-375.
2. Davani-Davari D, Karimzadeh I, Khalili H. The potential effects of anabolic-androgenic steroids and growth hormone as commonly used sport supplements on the kidney: a systematic review. *BMC Nephrol*. 2019; 20(1): 198.

### A8 - New equation to estimate the percentage of body fat in Colombian athletes

#### Santiago Gómez Velasquez^1^, Luis F. Bedoya Bedoya^1^, John E. Giraldo-Vallejo^1,2^, Gabriela Giraldo-Martínez^1^, Jacobo Martínez^1,2^, Katherine Franco-Hoyos^1^

##### ^1^Grupo de investigación NUTRAL, Facultad Ciencias de la Nutrición y los Alimentos, Universidad CES, Medellín, Colombia; ^2^Research Division, Dynamical Business & Science Society – DBSS International SAS, Bogotá, Colombia

###### **Correspondence:** Santiago Gómez Velasquez (sagomez@ces.edu.co)

*BMC Proceedings 2024*, **18(Suppl 4)**A8

**Introduction:** Anthropometry-based equations may help overcome limitations associated with reference methods such as Dual-Energy X-ray Absorptiometry (DXA), particularly in terms of cost, logistics, handling, in-field applications, and data interpretation in different sports and clinical contexts [1-3]. The aim of this study was to develop and validate an anthropometric equation to estimate the percentage of body fat (%BF) in the Colombian athlete population. **Methods:** A cross-sectional study was conducted based on the Standardized Reporting of Secondary Data Analyses (STROSA) guidelines, using DXA as the reference method. The data correspond to 132 records collected conveniently from 2019 to 2022. All measurements were taken on Colombian athletes at the Center for Advanced Studies in Nutrition and Food – CESNUTRAL at Universidad CES (Medellín, Colombia). For the development of the new equation, a sample of 100 participants was randomly assigned to the development group. Estimation models were constructed using multiple linear regression and the subset selection method to exclude irrelevant predictors. A validation sample of the remaining 32 participants was used to validate the selected model. Concordance analyses were performed using Bland-Altman plots. **Results:** The model that demonstrated higher performance (R^2^ = 0.80) included the variables abdominal skinfold, triceps skinfold, and Thigh 1 cm gluteal (maximum thigh) girth. The new equation is expressed as %BF = Abdominal Skinfold × 0.29 + Triceps Skinfold × 0.36 + Thigh 1 cm gluteal Girth × 0.21 – 7.65. **Conclusions:** A new equation was developed to estimate the percentage of body fat in Colombian athletes, showing a moderate-to-high correlation and high concordance when compared to measurements obtained by DXA.

**References**

1. Pruna R, Lizarraga A, Domínguez D. Medical assessment in athletes. *Med Clin (Barc)*. 2018; 150(7): 268-274.
2. Kasper AM, Langan-Evans C, Hudson JF, Brownlee TE, Harper LD, Naughton RJ, Morton JP, Close GL. Come Back Skinfolds, All Is Forgiven: A Narrative Review of the Efficacy of Common Body Composition Methods in Applied Sports Practice. *Nutrients*. 2021; 13(4): 1075.
3. Ackland TR, Lohman TG, Sundgot-Borgen J, Maughan RJ, Meyer NL, Stewart AD, Müller W. Current status of body composition assessment in sport: review and position statement on behalf of the ad hoc research working group on body composition health and performance, under the auspices of the I.O.C. Medical Commission. *Sports Med*. 2012; 42(3): 227-249.

